# Anti-inflammatory properties of shikonin contribute to improved early-stage diabetic retinopathy

**DOI:** 10.1038/srep44985

**Published:** 2017-03-21

**Authors:** Po-Lin Liao, Cheng-Hui Lin, Ching-Hao Li, Chi-Hao Tsai, Jau-Der Ho, George C. Y. Chiou, Jaw-Jou Kang, Yu-Wen Cheng

**Affiliations:** 1 Institute of Toxicology, College of Medicine, National Taiwan University, Taipei, Taiwan, ROC; 2 School of Pharmacy, College of Pharmacy, Taipei Medical University, Taipei, Taiwan ROC; 3 Department of Physiology, School of Medicine, College of Medicine, Taipei Medical University, Taipei, Taiwan, ROC; 4 Department of Pharmacology, School of Medicine, College of Medicine, Taipei Medical University, Taipei, Taiwan, ROC; 5 Department of Ophthalmology, Taipei Medical University Hospital, Taipei, Taiwan, ROC; 6 Institute of Ocular Pharmacology, College of Medicine, Texas A&M Health Science Center, College Station, TX, USA

## Abstract

Diabetic retinopathy (DR), a major microvascular complication of diabetes, leads to retinal vascular leakage, neuronal dysfunction, and apoptosis within the retina. In this study, we combined STZ with whole-body hypoxia (10% O_2_) for quicker induction of early-stage retinopathy in C57BL/6 mice. We also compared the effects of a high glucose condition combined with hypoxia (1% O_2_) to a low glucose condition by using retinal pigment epithelial (RPE) cells, which are a crucial component of the outer blood-retinal barrier and the damage is related to retinopathy. In the retina of DM/hypoxic C57BL/6 mice, abnormal a-wave and b-wave activity, yellowish-white spots, hyperfluorescence, and reduced retinal thickness were found using electroretinography (ERG), fundus photography (FP), fundus fluorescein angiography (FFA), and optical coherence tomography (OCT). Shikonin dose-dependently (0.5–50 mg/kg, *per os*) prevented DM/hypoxia-induced lesions. In eye tissue, administration of shikonin also attenuated DM/hypoxia-induced pre-apoptotic protein BAX expression as well as the production of inflammatory proteins cyclooxygenase-2 (COX-2) and inducible nitric oxide synthase (iNOS). We also demonstrated that shikonin administration rescues high glucose/hypoxia (1% O_2_)-induced inflammation, decreased junction protein expression, and permeability in RPE cells. These results indicate that shikonin treatment may prevent the loss of vision associated with DR.

More than 347 million people worldwide have diabetes, and it is expected to become the 7th leading cause of death by 2030^1^,^2^. Diabetic retinopathy (DR), a major microvascular complication of diabetes, is one of the leading causes of vision loss and visual impairment in the working age population worldwide^3^. The pathogenesis of DR is extremely complex since multiple cross-linked mechanisms are involved, leading to cellular dysfunction and adaptive changes in the retina^4^.

The clinical severity of DR ranges from nonproliferative (NPDR) and preproliferative (PDR) to more severely proliferative DR; total or partial vision loss can occur as a result of vitreous hemorrhage or retinal detachment. Central vision loss can also be a consequence of retinal vessel leakage and subsequent edema^5^,^6^. NPDR is characterized by the presence of microaneurysms and “dot and blot” hemorrhages, while PDR is characterized by abnormal retinal neovascularization. Clinically important consequences of PDR include retinal and vitreous hemorrhage, as well as tractional retinal detachment.

Most currently available treatments targeting DR neovascularization are invasive, including laser photocoagulation, intravitreal injection of anti-vascular endothelial growth factor (VEGF) agents or steroids, and vitrectomy^7^,^8^. In addition, these treatments are not effective in the early stages of DR, nor do they completely eliminate the risk of blindness^5^. Therefore, new treatment strategies are needed that are preventative and/or can be used earlier in the disease process to delay or prevent the progression of DR.

The exact mechanisms by which hyperglycemia initiates vascular and neuronal alterations in DR have not been completely elucidated^9^,^10^. The retinal lesions observed during early DR may be additive to, or synergistic with, proinflammatory changes, vaso-occlusion, and altered vascular permeability. Thus, the lesions may be intimately linked to the progressive ischemia and hypoxia that occur during the disease^9^,^11^.

Extensive research has been carried out using animal models of diabetes. This has provided valuable information regarding the cellular and molecular aspects of early-stage DR pathogenesis^12^,^13^,^14^,^15^. Several such models involve the chemical induction of diabetes using streptozotocin (STZ). Approximately six months after the onset of disease, STZ-induced diabetic C57BL/6 mice demonstrate early characteristics of DR such as acellular capillaries and vascular cell apoptosis in the retina^16^. In other studies, loss of ganglion cells and significant thinning of the inner and outer layers of the retina have been reported^17^,^18^. Taken together, these results indicate that STZ-induced diabetes is a suitable model of early-stage DR.

Shikonin is a major red naphthoquinone pigment that can be isolated from the root of *Lithospermum erythrorhizon* Sieb. et Zucc. (Boraginaceae) and has been used in traditional herbal medicine as an ointment for treating measles, sore throats, and burns^19^. Its chemical structure is shown in Fig. 1A.

Numerous studies have demonstrated that shikonin has a variety of clinical effects, including antimicrobial effects^20^,^21^, antioxidant effects^22^,^23^,^24^, and proliferative effects in wound healing^25^,^26^,^27^,^28^. It is an anti-allergenic agent^29^,^30^ and has anti-cancer effects^31^,^32^. Additionally, shikonin has been shown to be a potent anti-inflammatory agent^33^,^34^,^35^.

Given that there is a long onset period in mice (several months) for the induction of DR by STZ alone, we combined STZ induction of diabetes with systemic hypoxia (10% O_2_) to mimic the long-term inflammation and hypoxia that occur in early-stage DR^10^,^36^,^37^,^38^. The efficacy of shikonin for attenuating DR was then investigated. Potential targets of therapeutic application, including neuronal impairment and vascular permeability changes, were studied both *in vivo* and *in vitro*. With the aid of functional and molecular procedures, we show that mechanisms underlying the anti-DR effects of shikonin include anti-inflammatory and anti-apoptotic pathways, and that shikonin may contribute to the maintenance of normal vascular permeability.

## Results

### Animal data

**Figure 1:**
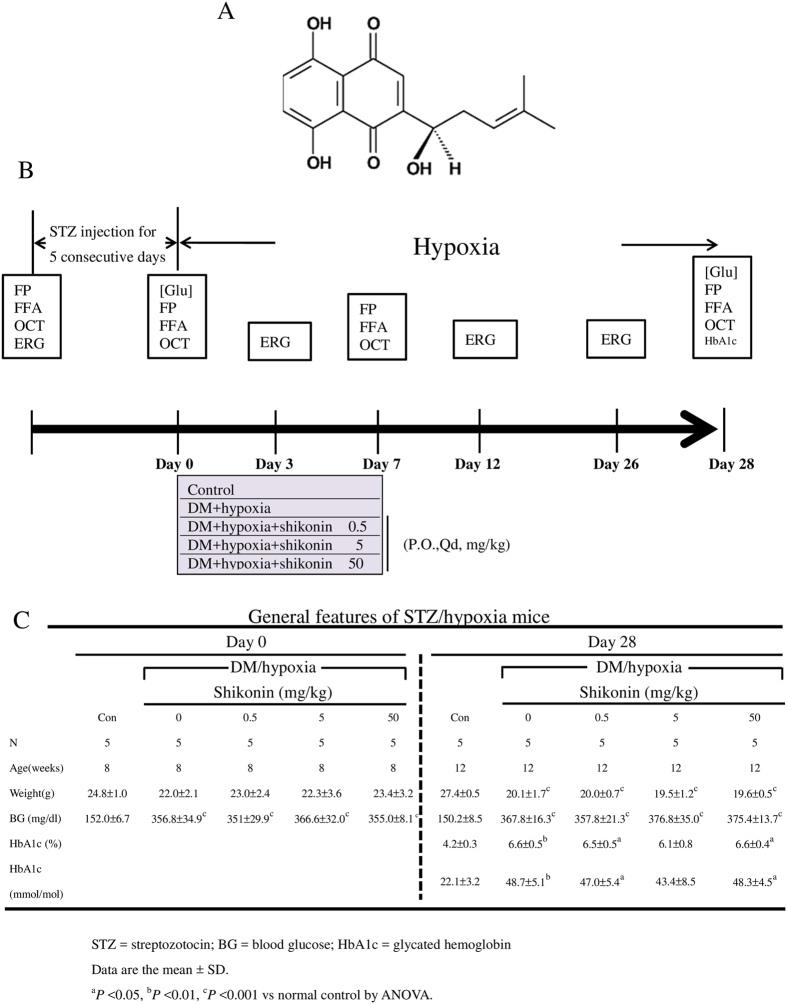
Development of diabetes in C57BL/6 mice treated with streptozotocin (STZ) and hypoxia. (**A**) Chemical structure of shikonin. (**B**) A schematic of STZ-induced diabetes combined with hypoxia is shown. Intraperitoneal (IP) injections of STZ (55 mg⁄kg) were given daily to C57BL/6 mice for five days. A control group was injected with an equivalent volume of citrate buffer only. After induction of diabetes, mice were randomly separated into five groups; a control group was placed under normoxic condition, whereas the other four groups were placed in a sealed hypoxia chamber and supplied with 10% oxygen. Three of the four hypoxia groups received 0.5 mg/kg, 5 mg/kg, or 50 mg/kg of shikonin daily *per os*. The final hypoxia group was given 1% DMSO only. Electroretinograms (ERG) were taken on days 3, 12, and 25, while fundus photography (FP), fundus fluorescein angiography (FFA), and optical coherence tomography (OCT) were performed weekly until day 28. Successful development of experimental diabetes in the STZ-treated mice was confirmed by blood glucose measurement at days 0 and 28. (**C**) General features of blood glucose and HbA1c levels in DM/hypoxic C57BL/6 mice treated with/without 0.5 mg/kg, 5 mg/kg, or 50 mg/kg of shikonin are presented. Data are expressed as mean ± SD of five animals per group. ^a^*p* < 0.05, ^b^*p* < 0.01, ^c^*p* < 0.001 vs normal control by ANOVA.

While blood glucose levels in control mice were 152 ± 6.7 mg/dL, mice in the DM/hypoxic group had significantly elevated levels of blood glucose (351 ± 29.9 ~ 366.6 ± 32 mg/dL, p < 0.001) after STZ injections, indicating successful induction of the diabetic model. Blood glucose remained >250 mg/dL (357.8 ± 21.3 ~ 376.8 ± 35 mg/dL, p < 0.001) on day 28 of the DM/hypoxic condition (Fig. 1C). DM/hypoxic mice and DM/hypoxic mice treated with 0.5 mg/kg, 5 mg/kg, or 50 mg/kg of shikonin had significantly lower body weights than age-matched healthy mice (Fig. 1C).

### ERG examination of the effects of shikonin on DM/hypoxia-induced retinopathy

**Figure 2:**
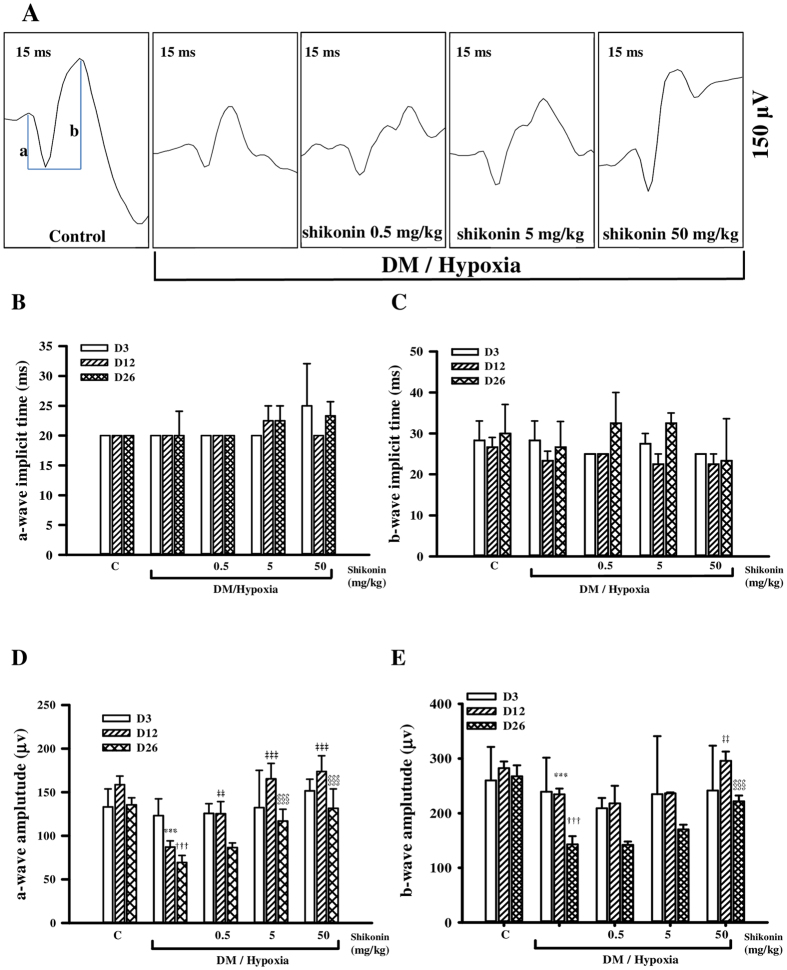
Electroretinogram (ERG) recordings in dark-adapted C57BL/6 mice. (**A**) Representative diagrams of a-wave and b-wave from the control groups, untreated DM/hypoxic groups, and the three shikonin-treated DM/hypoxic groups (various doses) on day 26. ERG data were collected on days 3, 12, and 26, and the mean implicit times (ms) of a- and b-waves are shown in (**B** and **C**) respectively. Amplitudes (μV) of a- and b-waves in the ERG waveform were also analyzed by histogram and are shown in (**D** and **E**), respectively. Data are expressed as mean ± SD from five animals. ***p < 0.001 compared with the day 12 control group. ^‡‡^*p* < 0.01, ^‡‡‡^*p* < 0.001 compared with the day 12 DM/hypoxic group. ^†††^ < 0.001 compared with the day 26 control group. ^§§§^*p* < 0.001 compared with the day 26 DM/hypoxic group.

An ERG is a diagnostic test that measures the electrical activity generated by both neuronal and non-neuronal cells in the retina in response to a light stimulus. We performed ERG examinations on days 3, 12, and 26 after initiating hypoxia. A representative ERG from day 26 is shown in Fig. 2A. The implicit times of a-waves and b-waves were not significantly different among the groups (Fig. 2B and C). The mean a-wave amplitude in the DM/hypoxia-induced mice was significantly smaller than the control group, both at day 12 (86.9 ± 8.2; *p* < 0.001) and at the end of the study (69.2 ± 8.2; *p* < 0.001). As shown in Fig. 2D, shikonin dose-dependently reversed the reduction in a-waves associated with DM/hypoxia. In addition, the mean amplitude of b-waves in DM/hypoxia-induced DR mice was 234.7 ± 10.3 at day 12 and 143 ± 14.9 at day 26, both of which were lower than the amplitude of b-waves in controls; however, shikonin administration dose-dependently reversed this detrimental effect (Fig. 2E).

### FP and FFA analysis of the effects of shikonin on DM/hypoxia-induced retinopathy

**Figure 3:**
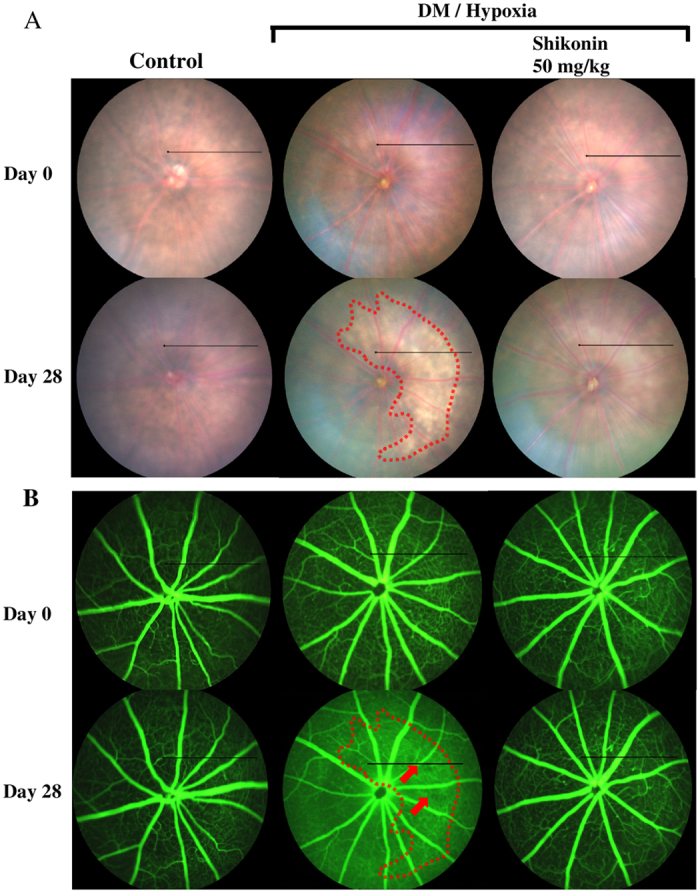
Ocular fundus images and fluorescein angiography *in vivo* analysis. (**A**) Fundus images were taken weekly, and images from the control group, the untreated DM/hypoxic groups, and the 50 mg/kg shikonin treatment group at days 0 and 28 are represented. The area surrounded by the dotted line represents geographic lesions in the retinas of the untreated DM/hypoxic group at day 28. (**B**) Fluorescein angiography was performed approximately 30 seconds after intravenous injection of a 10% sodium fluorescein solution. The area in the dotted line shows a hyperfluorescent lesion in retina and red arrows indicate several hypofluorescent areas in the retina of untreated diabetic/hypoxic mice at day 28. Black lines from (**A**) and (**B**) indicate the region selected for vertical scan OCT in Fig. 4.

After the first FP and FFA, the treatment groups were placed into a whole-body hypoxia chamber and orally administered 0.5 mg/kg, 5 mg/kg, or 50 mg/kg shikonin every day for 28 days. FP, FFA, and OCT examinations were performed weekly as shown in Fig. 1B. Figure 3A and B show representative FP and FFA micrographs from the control mice, untreated DM/hypoxic mice, and DM/hypoxic mice treated with 50 mg/kg shikonin on days 0 and 28. In healthy retina, FP and FFA results show a clear region of full fundi and fluorescent images. The fundi of DM/hypoxic mice showed yellowish-white spots, contiguous patches, and lesions reminiscent of geographic damage in all FPs taken from day 14 to day 28 (Fig. 3A, demarcated area). The area corresponding to the yellowish-white spots in the FP showed hyperfluorescence under FFA examination (Fig. 3B. demarcated area). Regional hypofluorescence was also observed (Fig. 3B, red arrows). In addition, the blurry whole-retina image from the untreated DM/hypoxic group indicates abnormal permeability of the vasculature to fluorescein. Notably, *QD*-treatment with 50 mg/kg shikonin successfully prevented geographic damage, hyperfluorescence, regional hypofluorescence, and the increased vascular permeability caused by DM/hypoxia-induced retinopathy.

### OCT analysis of the effects of shikonin on DM/hypoxia-induced retinopathy

**Figure 4:**
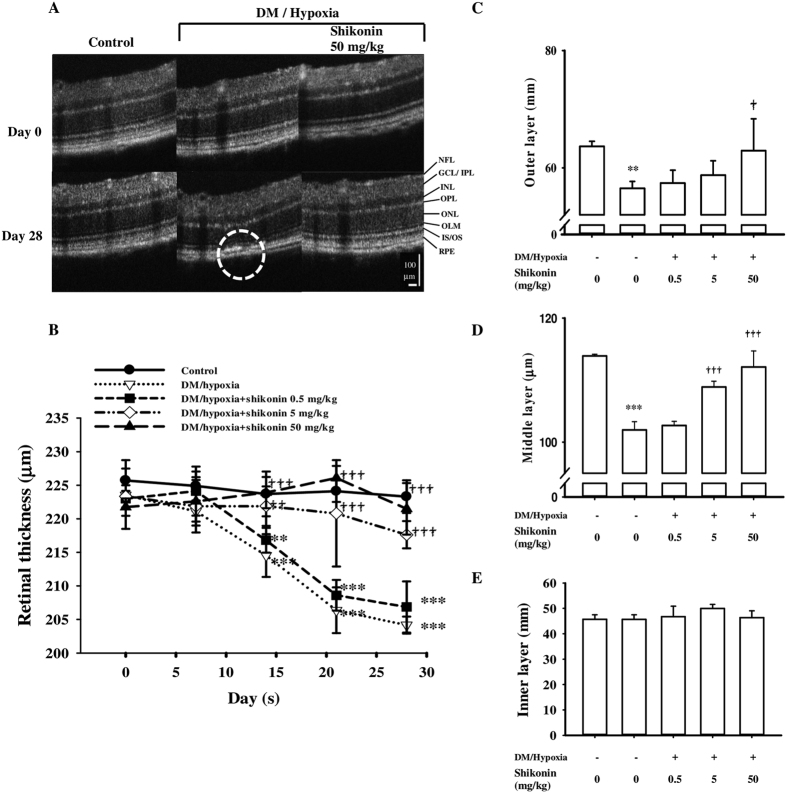
Spectral domain optical coherence tomography (SD-OCT) examination showed *in vivo* retinal alterations in C57BL/6 mice. (**A**) SD-OCT scan data from days 0 and 28 are presented. A definition of the retinal layers is shown on the right of the panel. OCT was performed in the same mice after FP and FFA, showing the retina in detail. The area inside the dotted line represents a geographic lesion in the retina of an untreated DM/hypoxic mouse on day 28. The arrow shows the retinal pigment epithelial (RPE) layer rupturing Bruch’s membrane. Weekly retinal total thickness measurements from SD-OCT in the control group, the untreated DM/hypoxic group, and the three shikonin treatment groups are presented in (**B**). Data are expressed as mean ± SD from 5 animals. **p < 0.01, ***p < 0.001 compared with the relative control. ^††^*p* < 0.01, and ^†††^*p* < 0.001 compared with the untreated DM/hypoxic group. Three layers from the retina of a mouse on day 28 are shown in our SD-OCT scan: the outer layer (NFL-IPL), the middle layer (IPL-IS/OS), and the inner layer (IS/OS-RPE) (**C**–**E**). Data are expressed as mean ± SD from 5 animals. **p < 0.01, ***p < 0.001 compared with the control group; ^†^*p* < 0.05, ^†††^*p* < 0.001 compared with the untreated DM/hypoxic group.

To further characterize the lesions observed by FP and FFA and to verify the efficacy of shikonin, we used real-time OCT to define FP and FFP regions (Fig. 3A and B, black lines). Representative OCT micrographs (Fig. 4A) were present on day 0 and day 28 from the control group, untreated DM/hypoxic group, and DM/hypoxic group receiving 50 mg/kg shikonin. The cross-sectional OCT b-scan clearly showed distinct retinal layers, including the NFL, GCL, IPL, INL, OPL, ONL, OLM, IS/OS of the photoreceptor layer, retinal pigment epithelium, and the choroid. These are indicated to the right of the panel (Fig. 4A). The retinas from DM/hypoxic mice that did not receive additional treatments showed abnormalities on day 14 that continued until the end of the experiment. On day 28, the retinal pigment epithelium was clearly detached (arrow) and structural disturbances in the architecture of the nuclear layers were found (demarcated area) (Fig. 4A). Shikonin prevented these DM/hypoxia-induced structural lesions. Retinal thickness was measured weekly. As shown in Fig. 4B, the retinal thickness of normal control mice ranged from 223.31 ± 2.4 ~ 225.72 ± 3.02 μm and the total thickness of the retinas from DM/hypoxic mice was reduced in a time-dependent manner to 214.52 ± 3.19 (day 14; *p* < 0.001), 206.36 ± 3.41 (day 21; *p* < 0.001), and 204.16 ± 1.26 (day 28; *p* < 0.001). Three layers of retina were defined as the inner layer (NFL-IPL), the middle layer (INL-OLM), and the outer layer (IS/OS-RPE). The thickness of each layer at day 28 in the control group, untreated DM/hypoxic group, and DM/hypoxic group treated with shikonin (0.5–50 mg/kg) is shown in Fig. 4C–E. We found that the reductions in retinal thickness observed in the DM/hypoxic mice occurred both in the middle photoreceptor and in the inner neuron layer.

### Histological examination of shikonin in DM/hypoxia-induced retinopathy

**Figure 5:**
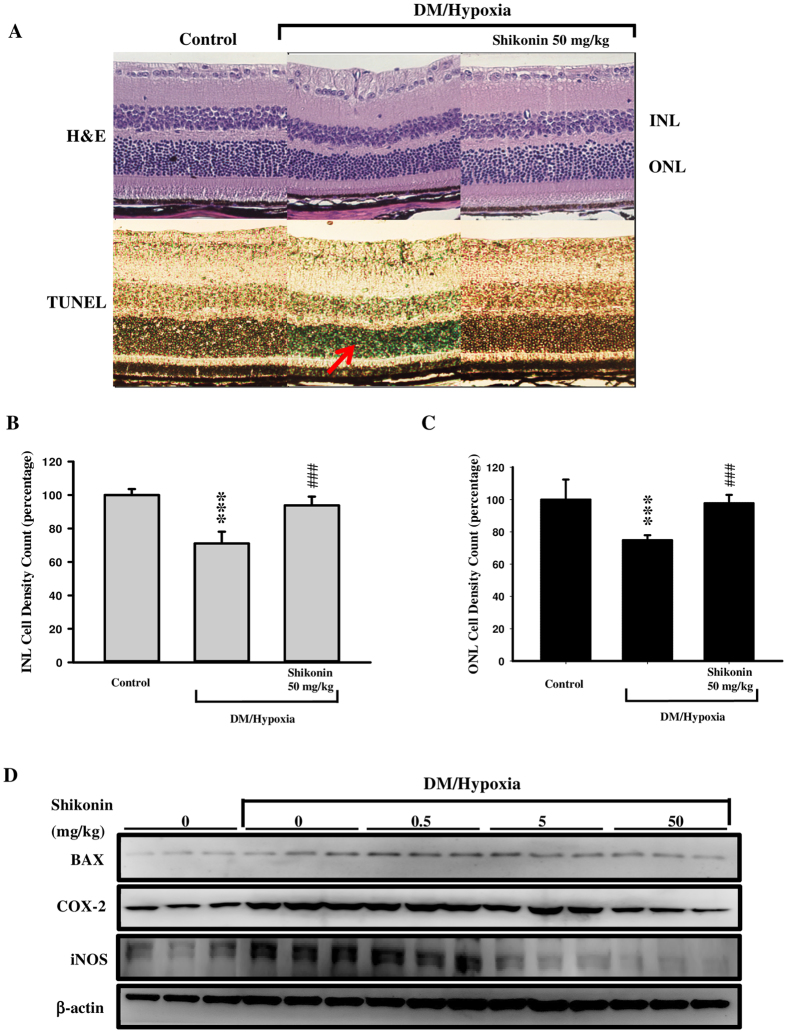
Histological examination of the effects of shikonin on streptozotocin (STZ)/hypoxia-induced diabetic retinopathy in C57BL/6 mice. (**A**) Morphology of the retina from the control group on day 28 (upper part of the picture), the untreated DM/hypoxic group, and the 50 mg/kg shikonin-treated group is shown by H&E staining. The lower panel shows the light blue, TUNEL-positive, cells in the retina from a representative untreated DM/hypoxic mouse (red arrow). (**B**) Inner nuclear layer (INL) cell count. (**C**) Outer nuclear layer (ONL) cell count. Data are expressed as mean ± SD from 5 animals. ***p < 0.001 compared with the control group; ^###^*p* < 0.001 compared with the untreated DM/hypoxic group. (**D**) The left eye of three randomly selected mice from each group (5 animals) was individually collected and subjected to tissue homogenization at high speed for 2 × 30 sec using a Minilys tissue homogenizer (Bertin Technologies, France). The homogenate was then centrifuged at 14,000 g for 10 min at 4 °C and the supernatant was collected. The protein concentration in the supernatant was determined with Bradford reagent. Absorbance at 595 nm was measured using a spectrophotometer (Optizen POP, Korea). Lysate proteins (80 μg) from each eye were analyzed by separation on 10% reducing SDS-PAGE gels and western blotting.

After the 28-day procedure in our DM/hypoxia-induced retinopathy model, all mice were euthanized and the right eye was stained with hematoxylin and eosin (H&E). As shown in Fig. 5A, structural disturbances and cell loss in the ONL, INL, and NFL were observed. However, administration of 50 mg/kg of shikonin rescued DM/hypoxia-induced abnormalities. To characterize cell loss observed in the ONL, INL, and NFL of DM/hypoxic mice, we used the terminal deoxynucleotidyl transferase-mediated dUTP nick end-labeling (TUNEL) assay. The retina of DM/hypoxic mice showed slight TUNEL-positive cells in the ONL, whereas the retina from control mice and DM/hypoxic mice receiving 50 mg/kg of shikonin treatment had no TUNEL positive cells. Cell count analysis was also performed. As shown in Fig. 5B and C, INL and ONL cells significantly decreased in the DM/hypoxic group, while oral administration of shikonin (50 mg/kg) rescued the cell loss. The left eye of three randomly selected mice from each group was collected and subjected to western blot analysis. We found that expression of the pre-apoptotic BCL-2 family protein (BAX), the inflammatory-related protein cyclooxygenase-2 (COX-2), and inducible nitric oxide synthase (iNOS) was higher in DM/hypoxic mice than controls; however, DM/hypoxic mice treated with shikonin had a dose-dependent reduction in the expression of these proteins, which returned to baseline (Fig. 5D). In combination with the corresponding FP, FFA, and OCT data, this histological analysis suggests that shikonin dose-dependently protects against lesion formation, RPE cell damage, vascular permeability, and possibly edema.

### Shikonin protects RPE cells from high-glucose/hypoxia-induced damage

**Figure 6:**
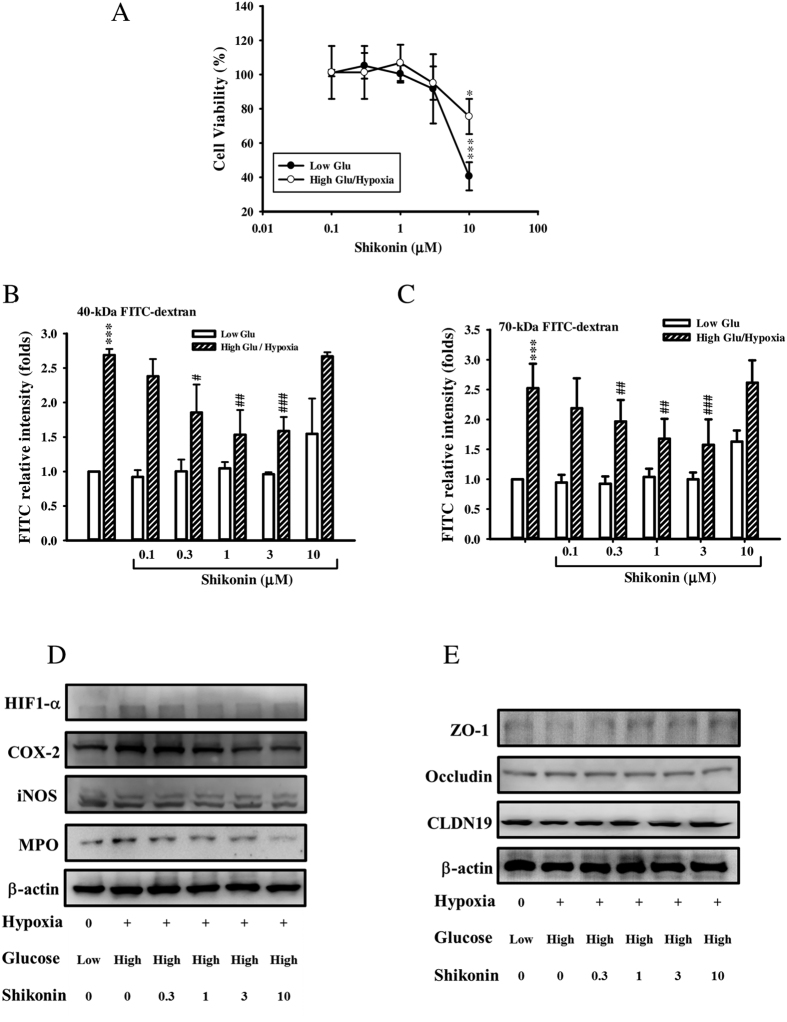
The effect of shikonin on high glucose/hypoxia damaged retinal pigment epithelial (RPE) cells. RPE cells incubated under high or low glucose concentrations and hypoxia (1% O_2_) with or without shikonin (0.01–10 μM) for 24 hr. Cell viability was compared between cells incubated under low or high glucose concentrations using the MTT assay. Data are expressed as mean ± S.D. from at least three independent experiments. *p < 0.05, ***p < 0.001 compared with low glucose control. (**A**) Paracellular tracer-flux assays were performed using FITC-dextran (molecular masses, 40 kDa (**B**) and 70 kDa (**C**), respectively) and dense RPE monolayers treated with or without shikonin (0.01–10 μM, 24 h). Results from at least 3 independent experiments are shown. ***p < 0.001 compared with low glucose control. ^#^*p* < 0.05, ^##^*p* < 0.01, and ^###^*p* < 0.001, compared with untreated high glucose/hypoxia group. (**D**) RPE cells were incubated with or without shikonin (0.003–0.3 μM) for 24 h under low-glucose/hypoxic or high-glucose/hypoxic conditions. Cell lysate proteins (40 μg) from each incubation were analyzed by separation on 10% reducing SDS-PAGE gels and western blotting with specific antibodies against inflammatory proteins hypoxia inducible factor 1-α (HIF1-α), cyclooxygenase-2 (COX-2), inducible nitric oxide synthase (iNOS), and myeloperoxidase (MPO). (**E**) Representative images of western blots showing changes in the expression of adherens junction proteins and tight junction proteins in RPE cells treated with/without shikonin under low-glucose/hypoxic or high-glucose/hypoxic conditions.

RPE cells are a crucial component of the outer blood-retinal barrier; the progression of diabetic retinopathy is associated with damage to RPE cells. The hyperfluorescence observed in DM/hypoxic mice under FFA examination (Fig. 3B) may be a result of damage to RPE cells. Here, we investigated several pro-inflammatory and junction proteins, as well as cell permeability in RPE cells. Different concentrations of shikonin (0.1–10 μM) were added to RPE cultures incubated in low-glucose (1 g/L) or high-glucose (4.5 g/L) DMEM with 10% heat-inactivated FBS. After 30 min, the high-glucose treated RPE cells were incubated in a hypoxia chamber (1% O_2_) for 24 h, and then their viability was measured by MTT assay. As shown in Fig. 6A, cell viability remained unaffected in cultures treated with concentrations of shikonin less than 3 μM, while 10 μM of shikonin caused cytotoxicity in both the low-glucose and high-glucose/hypoxia treated RPE cells. Paracellular permeability of low-glucose and high-glucose/hypoxia treated RPE cells incubated with shikonin (0.3–10 μM) was measured using FITC-labeled dextran. A marked increase in the flux of 40- and 70-kDa FITC-dextran was observed in the high-glucose/hypoxia group (Fig. 6B,C), while shikonin (0.3–3 μM) concentration-dependently decreased this flux. However, the increased flux in 40- and 70-kDa FITC-dextran was again observed in RPE cells treated with 10 μM of shikonin. Next, we performed western blotting to analyze changes in the expression of inflammatory and junction proteins. As shown in Fig. 6D, high-glucose/hypoxia enhanced the expression of hypoxia-inducible factor 1-α (HIF 1-α), COX-2, and myeloperoxidase (MPO). Shikonin (0.3–3 μM) concentration-dependently reduced these increases in protein expression. Interestingly, expression of iNOS was unaffected by high-glucose/hypoxia in RPE cells. In contrast, expression of the tight junction protein, ZO-1, was decreased by high-glucose/hypoxia, an affect that was attenuated in a concentration-dependent manner by treatment with shikonin (0.3–10 μM). Occludin and claudin-19 levels remained unchanged for all treatments (Fig. 6E).

## Discussion

A significant health burden attributable to diabetic complications persists because of a continuing increase in the prevalence of diabetes, along with poor glycemic control in the early stages of disease. Recent studies have shown that early glucose control does not prevent DR^39^; thus, significant efforts are needed to find new therapies for DR.

To date, there is no perfect animal model that recapitulates all aspects of human DR, and lesion onset times vary between species and strains. Specifically, 4–8 months after induction of diabetes, Lewis rats show the fastest loss of retinal capillaries and retinal ganglion cells (RGCs) of all models, Wistar rats show degeneration of the capillaries without significant neurodegeneration, and Sprague Dawley rats show no lesions^39^. STZ-induced diabetic B6 mice also demonstrate early characteristics of DR approximately 6 months after the onset of diabetes^16^.

In our study, we evaluated the effects of the anti-inflammatory agent, shikonin, on DM/hypoxia–induced DR. Vascular and non-vascular alterations of DR were monitored in the same animals in real-time by using ERG, FP, FFA, and OCT. Here, lesions indicative of early-stage DR were found as early as 12 days after initiating DM/hypoxic conditions. All defined lesions were confirmed by Jau-De, Ho, M.D., Chief of Ophthalmology, Taipei Medical University Hospital. As retinal neurodegeneration is an early event in the pathogenesis of DR, ERG was performed. The a-wave represents the initial corneal-negative deflection deriving from the cones and rods of the outer photoreceptor layers, while the b-wave indicates the corneal-positive deflection originating from the inner neuron functions of the retina. Our results show that untreated DM/hypoxic mice had significantly lower a-wave and b-wave amplitudes (Fig. 2D,E), indicating injury to the photoreceptors in the outer retinal cells and retinal inner layers, respectively. Indeed, results from OCT scans indicated that total retinal thickness of C57BL/6 mice began to significantly decrease two weeks after initiating diabetes/hypoxia (Fig. 4B). Shrinkage of the middle retinal layer is the major cause of the decline in retinal thickness, while the inner layer is partially involved (Fig. 4C,D). Finally, morphological observations by H&E staining and detection of DNA damage by TUNEL staining further confirmed these results by indicating the presence of apoptotic cells (Fig. 5A). Yellowish-white spots and lesions reminiscent of geographic atrophy observed in FP and corresponding hyperfluorescent areas observed by FFA showed that retinal pigment epithelial impairment and increased retinal vascular permeability had occurred two weeks after the start of DM/hypoxia treatment in C57BL/6 mice (Fig. 3A,B). Several hypofluorescent spots were also found, suggesting that regional edema may have occurred. In addition, we assessed normal C57BL/6 mice under hypoxic conditions as well as STZ-induced diabetic C57BL/6 mice using the same experimental tools and the same experimental timeline as described above, and found no visible lesions (data not shown); this indicates that neither diabetes nor hypoxia alone was sufficient to induce the rapid onset of lesions observed in the C57BL/6 mice used in this study.

Hypoxia, resulting in progressively worsening retinal ischemia, commonly causes macrophages and other immune cells to accumulate^40^. Activation of these cells results in the production of cytokines that induce the expression of proinflammatory proteins and results in increased apoptosis. Specifically, increased retinal expression of iNOS and COX-2 is known to be a key factor responsible for diabetes-induced retinal inflammation^36^. Here, eye tissues taken from DM/hypoxic mice showed a marked increase in the expression of iNOS and COX-2, as well as the pro-apoptotic protein, BAX (Fig. 5D).

We have demonstrated that an anti-inflammatory agent, shikonin, reduced the lesions in retinal tissues of DM/hypoxic mice. In DM/hypoxic mice given 50 mg/kg of shikonin, DR did not develop until the end of the experimental period (day 28). In contrast, DR-like lesions developed earlier in untreated DM/hypoxic mice. In addition, shikonin significantly attenuated the loss of neuron cells, as well as the number of retinal vascular leakages observed in DM/hypoxic mice. Since RPE cells are a crucial component of the outer blood-retinal barrier and their damage is related to the progression of diabetic retinopathy^41^, we investigated the effects of shikonin treatment on cultured high-glucose/hypoxia damaged RPE cells. We found that shikonin treatment exert a beneficial effect on cell permeability and expression of related junction proteins, as well as that of the inflammatory proteins HIF-1α, COX-2, and MPO (Fig. 6B–E). However, shikonin did not decrease the elevated flux of FITC-dextran attributable to high-glucose/hypoxic condition at 10 μM despite it recovered high-glucose/hypoxia-decreased ZO-1 expression. We predict that at this concentration, shikonin exerts some cytotoxic effect (Fig. 6A) and may alter other adherens and tight junction members, resulting in increased permeability. Notably, iNOS expression was unchanged in RPE cells following treatment, indicating that the increases in iNOS found during the *in vivo* experiments are likely attributable to other cells, such as glial cells.

We used DMSO as a solvent in our study; DMSO has recently been reported to exert anti-inflammatory effects on its own^42^,^43^. However, the amount of DMSO used in the present study was much smaller than that previously reported to have anti-inflammatory effects, and no beneficial effects were observed in DM/hypoxic mice treated with DMSO only, indicating that the ameliorative effects on DR were attributable to shikonin treatment and not DMSO. We also administered metformin, commonly used as a standard antidiabetic drug to compare hypoglycemic efficacy in STZ-induced models of moderate diabetes, to hypoxic/diabetic mice for 4 weeks by gavage. While metformin (300 mg/kg, q.d.) treatment significantly reduced fasting blood sugar, it failed to prevent DM/hypoxia-induced lesions as determined by FP and FFA observations (see Supplementary Fig. S1). Also, shikonin treatment had no effect on blood glucose concentrations (Fig. 1C), and weight loss was comparable to that of untreated DM/hypoxic mice, suggesting shikonin exerts unique effects in DR independent of blood glucose regulation. Although shikonin at 10 μM caused cytotoxicity in RPE cells in our *in vitro* experiments, no noticeable side effects were observed in DM/hypoxic mice administered 50 mg/kg shikonin daily. To ensure the safety of shikonin at the dosage we used in our experiments, serum glutamic oxaloacetic transaminase (GOT), glutamic pyruvic transaminase (GPT), and blood urea nitrogen (BUN) parameters were examined in mice after 28 days of daily treatment with 50 mg/kg shikonin, as well as in the control group. There were no observed adverse effects in the normal, DM/hypoxic, and shikonin-treated DM/hypoic C57Bl/6 mice (see Supplementary Table S1). Also, the repeated-dose chronic toxicity of shikonin derivatives was recently investigated in Wistar rats^44^; 800 mg/kg of shikonin given by daily gavage for 180 days did not cause hematological or non-hematological toxicity, indicating the therapeutic window for DR is large.

In conclusion, we have established a new DR animal model and successfully used high-resolution real-time fundus images to analyze the early-stages of a DR-like syndrome. These data suggest that shikonin is a promising therapeutic agent for DR. Further study is indicated to establish its clinical feasibility.

## Methods

A description of chemicals and reagents, paracellular permeability assay, cell culture and hypoxia treatment, histochemistry, TUNEL assay, and western blot analysis are provided in the Supplementary appendices.

### DM/hypoxia-induced DR model in C57BL/6 mice

An experimental flow chart is shown in Fig. 1B. All experimental procedures involving the use of animals complied with the Association for Research in Vision and Ophthalmology (ARVO) statements for the use of animals in ophthalmic and vision experimental research, and the animal use protocol listed below was reviewed and approved by the Institutional Animal Care and Use Committee of Taipei Medical University (approval number: LAC-2014-0194).

C57BL/6 mice (8 weeks old) were treated with 55 mg/kg STZ through daily intraperitoneal (IP) injection for 5 days and the non-diabetic control mice were injected with citrate vehicle only. Tail blood samples were then collected from fasting animals and analyzed for serum glucose levels using a Fuji Dri-Chem Slide and Fuji Dri-Chem 1000 (Fuji Film Med. Co., Tokyo, Japan) to verify successful development of experimental diabetes in the STZ-treated animals. C57BL/6 mice were then randomly separated into five groups, including a non-diabetic control group maintained under normoxic conditions. The four other diabetic groups were maintained in a hypoxia chamber supplied with 10% oxygen for 4 weeks (except for periods of drug treatment or performance of functional assays); three of the four groups were orally administered daily doses of 0.5 mg/kg shikonin, 5 mg/kg shikonin, or 50 mg/kg shikonin (dissolved in 1% dimethyl sulfoxide [DMSO]). The final group received hypoxia-treatment alone with an equivalent amount of 1% DMSO. The oxygen concentration was continuously monitored by a gas analyzer (Anaerobic System ProOx model 110; BioSpherix, Lacona, NY, USA). HbA1c (glycated hemoglobin) was determined by the Department of Laboratory Medicine, Taipei Medical University using cardiac blood collected when the animals were sacrificed.

### Fundus photography (FP) and fundus fluorescein angiography (FFA)

A Micron III retinal imaging microscope (Phoenix Research Laboratories, Pleasanton, CA) was used to monitor morphological and pathological changes in the fundus of C57BL/6 mice. Briefly, mice were anesthetized by IP injection of ketamine (80 mg/kg) and xylazine (10 mg/kg), and eyes were dilated with 0.125% atropine. Each mouse was held on its side on the microscope platform and the right eye was rinsed with 2% Methocel gel (OmniVision, SA, Neuhausen, Switzerland). After color FP was performed, fluorescein (10%; 0.05 mL) was used for FFA examination through IP injection. Serial images were then collected using SteamPix 5™ software.

### Optical coherence tomography (OCT) imaging and thickness analysis

The OCT module of the Micron III (Phoenix Research Laboratories, Pleasanton, CA) retinal imaging microscope was used to obtain images from retinal layers. A high-resolution b-scan of retinal cross-sections (right eye) was obtained by averaging and spatially aligning 5 individual b-scans along the same vertical axis. Retinal layers were segmented using InSight XL (Phoenix Research Laboratories, San Ramon, CA, SA) for further analysis. Three retinal layers were defined and measured in the C57BL/6 mice included in this study: the inner layer, which comprises the retinal nerve fiber layer (RNFL), the ganglion cell layer (GCL), and the inner plexiform layer (IPL); the middle layer, which comprises the inner nuclear layer (INL), the outer plexiform layer (OPL), the outer nuclear layer (ONL), and the outer limiting membrane (OLM); and the outer layer, which comprises the inner and outer segments (IS/OS) of the photoreceptors and the retinal pigment epithelium.

### Electroretinogram (ERG)

A light stimulator (Grass PS33 Photic Stimulator; Grass Instruments, Warwick, U.S.A.) was held 10 cm from the right eye and allowed to flash before recording the ERG using Dawson, Trick, and Litzkow (DTL) fiber electrodes. ERG signals were amplified with an MP36 4-channel data acquisition system (BIOPAC Systems, Inc., Pershore, UK). ERG signals were amplified (DC to 300 Hz) and digitized at 1 kHz with a resolution of 2 μV. Wave amplitude was measured from the baseline to the trough of a- and b- waves. Implicit time is a measure of the time interval from the onset of the stimulus to the peak of the a- and b-wave.

### Statistical analysis

All data are expressed as the mean ± SD from at least 3 independent experiments (n ≥ 3). Statistically significant differences between groups were determined using one-way analysis of variance (ANOVA). A *p*-value < 0.05 was considered statistically significant.

## Additional Information

**How to cite this article:** Liao, P.-L. *et al*. Anti-inflammatory properties of shikonin contribute to improved early-stage diabetic retinopathy. *Sci. Rep.* **7**, 44985; doi: 10.1038/srep44985 (2017).

**Publisher's note:** Springer Nature remains neutral with regard to jurisdictional claims in published maps and institutional affiliations.

## Supplementary Material

Supplementary Information
