# Supplementary material for: Anti-inflammatory properties of shikonin contribute to improved early-stage diabetic retinopathy
Source: Sci Rep. 2017 Mar 21;7:44985. doi: 10.1038/srep44985 (PMC5359562; doi:10.1038/srep44985)
Supplement: Supplementary Information [file srep44985-s1.pdf]

Anti-inflammatory properties of shikonin contribute to improved early-stage diabetic retinopathy

Po-Lin Liao[a,b], Cheng-Hui Lin[b], Ching-Hao Li[c,d], Chi-Hao Tsai[a], Jau-Der Ho[e], George C.Y. Chiou[f], Jaw-Jou Kang[a,*] and Yu-Wen Cheng[b,*]

[a]*Institute of Toxicology, College of Medicine, National Taiwan University, Taipei, Taiwan, R.O.C.*

[b]*School of Pharmacy, College of Pharmacy, Taipei Medical University, Taipei, Taiwan R.O.C.*

[c]*Department of Physiology, School of Medicine, College of Medicine, Taipei Medical University, Taipei, Taiwan, R.O.C.*

[d]*Department of Pharmacology, School of Medicine, College of Medicine, Taipei Medical University, Taipei, Taiwan, R.O.C.*

[e]*Department of Ophthalmology, Taipei Medical University Hospital, Taipei, Taiwan, , R.O.C.*

[f]*Institute of Ocular Pharmacology, College of Medicine, Texas A&M Health Science Center, College Station, TX, USA*

[*] Equal contribution Corresponding authors

Corresponding author 1: Yu-Wen Cheng

Tel.: +886 2 27361661 x 6123

fax: +886-2-27374622.

E-mail address: ywcheng@tmu.edu.tw (Y.W. Cheng).

Corresponding author 2: Jaw-Jou Kang

Tel.: +886 2 23123456 x 88604

fax: +886-2-27361661

E-mail address: jjkang@ntu.edu.tw (J.J. Kang).

Supplementary Information

Chemicals and Reagents

Fetal bovine serum (FBS), Dulbecco's modified Eagle's medium (DMEM), penicillin/streptomycin/glutamate and trypsin-EDTA were obtained from Gibco BRL (Grand Island, NY, USA). iNOS Ab, COX-2 Ab were from Cell Signaling (Beverly, MA, USA). ZO-1 Ab, HIF-1α Aβ, myeloperoxidase (MPO) Ab, occluding Ab and claudin 19Ab were obtained from GeneTex, Inc. (TX, USA). BAX Ab was obtained from Santa Cruz Biomtechnology (Santa Cruz, CA, USA). The horseradish peroxidase (HRP)-conjugated anti-mouse immunoglobulin G (IgG) Ab and HRP-conjugated anti-rabbit IgG Ab were obtained from Amersham Biosciences (Sunnyrale, CA, USA). 2% pilocarpine hydrochloride (ISOPTOCARPINE®) was purchased from Alcon® Taiwan (Taipei, Taiwan). 0.125% atropine surfate (KINTROPINE®) was purchased from Synpac-Kindom Pharmaceutical Co., Ltd. (Taipei, Taiwan). Tetramethylbenzidine (TMB) substrate was purchased from Bethyl Laboratories Inc. (TX, USA). Other reagents and chemicals including shikonin were obtained from Sigma (St. Louis, MO, USA).

Cell culture and hypoxia treatment

Human retinal pigment epithelial (RPE) cells were obtained from American Type Culture Collection (Manassas, VA, USA). RPE cells were cultured in DMEM High glucose (4.5 g/L) or DMEM Low glucose (1 g/L) medium supplemented with 10% heat-inactivated fetal bovine serum (FBS) in a humidified atmosphere of 5% CO_2 at 37°C. To mimic hypoxia, RPE cells incubated in the same DMEM media were treated with/without shikonin for 30 min, and then conditioned in a hypoxia chamber with 95% N2 and 5% CO2 at 37°C for 24 hrs. (1 % O_2, Anaerobic System ProOx model 110, BioSpherix, Lacona, NY, USA; [1] .

Paracellular permeability assay

1×10^5 RPE cells were seeded on fibronectin pre-coated Transwell inserts (Corning, Cambridge, MA; pore size, 0.4 μm) and cultivated in DMEM of different glucose concentrations for 2–4 days until a dense epithelial monolayer had formed. Cells were then maintained under hypoxia for 24 h and subsequently treated with shikonin for 30 min. FITC-dextran (200 μg/mL; molecular mass, 40 or 70 kDa) was also added to the inner chamber. After 30 minutes, we measured the concentration of FITC-dextran in the outer chamber at (excitation wavelength, 485/535 nm [Abs/Em]) using a Paradigm Multi-Mode Plate Reader (Beckman Coulter Inc., Brea, CA).

Histochemistry

All C57BL/6 mice were sacrificed by ketamine/xylazine overdose after 28 days of DM/hypoxia. Eyes were collected and the anterior parts and lens were removed. To fix the eyecups for paraffin sectioning, we used modified Davidson's fixative [2] overnight. After being dehydrated in ethanol, they were embedded in paraffin. Radial 5-μm sections were collected and stained using hemotoxylin and eosin (H&E).

TUNEL assay

Apoptosis was detected by TUNEL (Terminal deoxynucleotidyl transferase-mediated dUTP Nick End-Labeling) assay in whole-mount eyecup sections using the "in situ cell death detection kit, POD" (Roche, Germany) according to the manufacturer's instructions.

Western blot analysis

RPE cells incubated in different glucose concentrations of DMEM were treated with or without shikonin under hypoxic conditions for 24 hr; the left eye of all experimental mice was collected immediately after sacrifice on the 28th day after DM/hopoxia induction. Eye tissues were then homogenized at high speed for 2 × 30 sec using a Minilys tissue homogenizer (Bertin technologies, France). The

homogenate from cells or tissues was then centrifuged at 14,000 g for 10 min at 4°C and the supernatant was collected. Proteins were separated by 10% reducing SDS-PAGE, electrotransferred onto polyvinylidene difluoride (PVDF) and was immunoblotted with antibodies against inflammatory, junction, and apoptotic proteins, as well as β-actin as a control.

References

1 Frank, R. N. Diabetic retinopathy. *The New England journal of medicine* **350**, 48-58, doi:10.1056/NEJMra021678 (2004).

2 van Eeden, P. E. *et al.* Early vascular and neuronal changes in a VEGF transgenic mouse model of retinal neovascularization. *Investigative ophthalmology & visual science* **47**, 4638-4645, doi:10.1167/iovs.06-0251 (2006).

Supplementary Table S1

	Day 0			Day 28		
		DM/hypoxia			DM/hypoxia	
Shikonin (mg/kg)	0	0	50	0	0	50
N	3	3	5	3	3	5
Age(weeks)	8	8	8	12	12	12
GOT(U/l)	75.3±10.7	75.0±5.2	72.0.±7.7	76.3±7.0	83.7±11.2	78.0±7.3
GPT(U/l)	45.0±5.2	42.3±3.1	46.8±5.4	40.7±6.0	48.3±3.1	50.0±4.2
BUN (mg/dL)	28.1±1.1	27.1±5.0	29.9±2.5	28.6±3.5	29.3±3.7	30.1±3.8

GOT = Glutamic oxaloacetic transaminase; GPT = Glutamic pyruvic transaminase; BUN = Blood urea nitrogen. Data are means ± SD.

Supplementary Figure S1

(A)

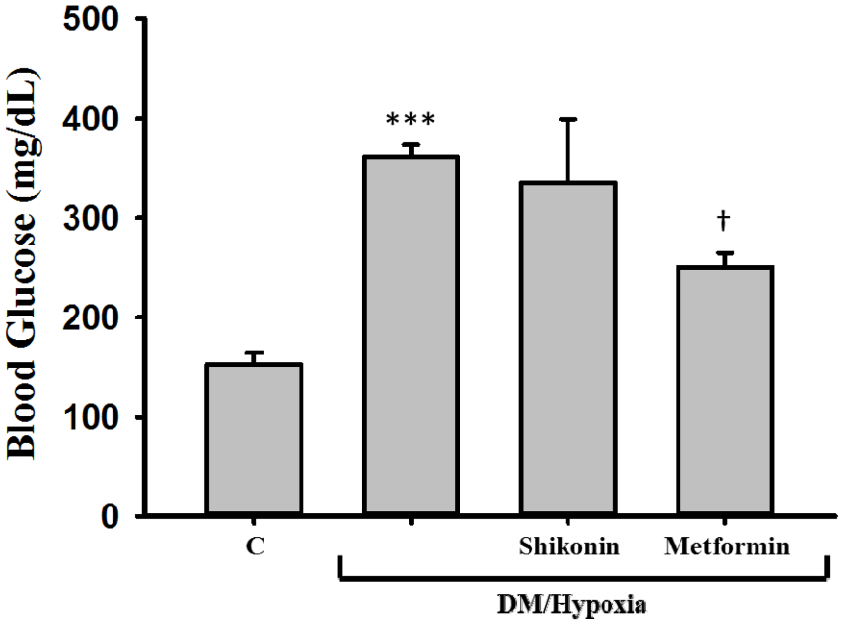

(B)

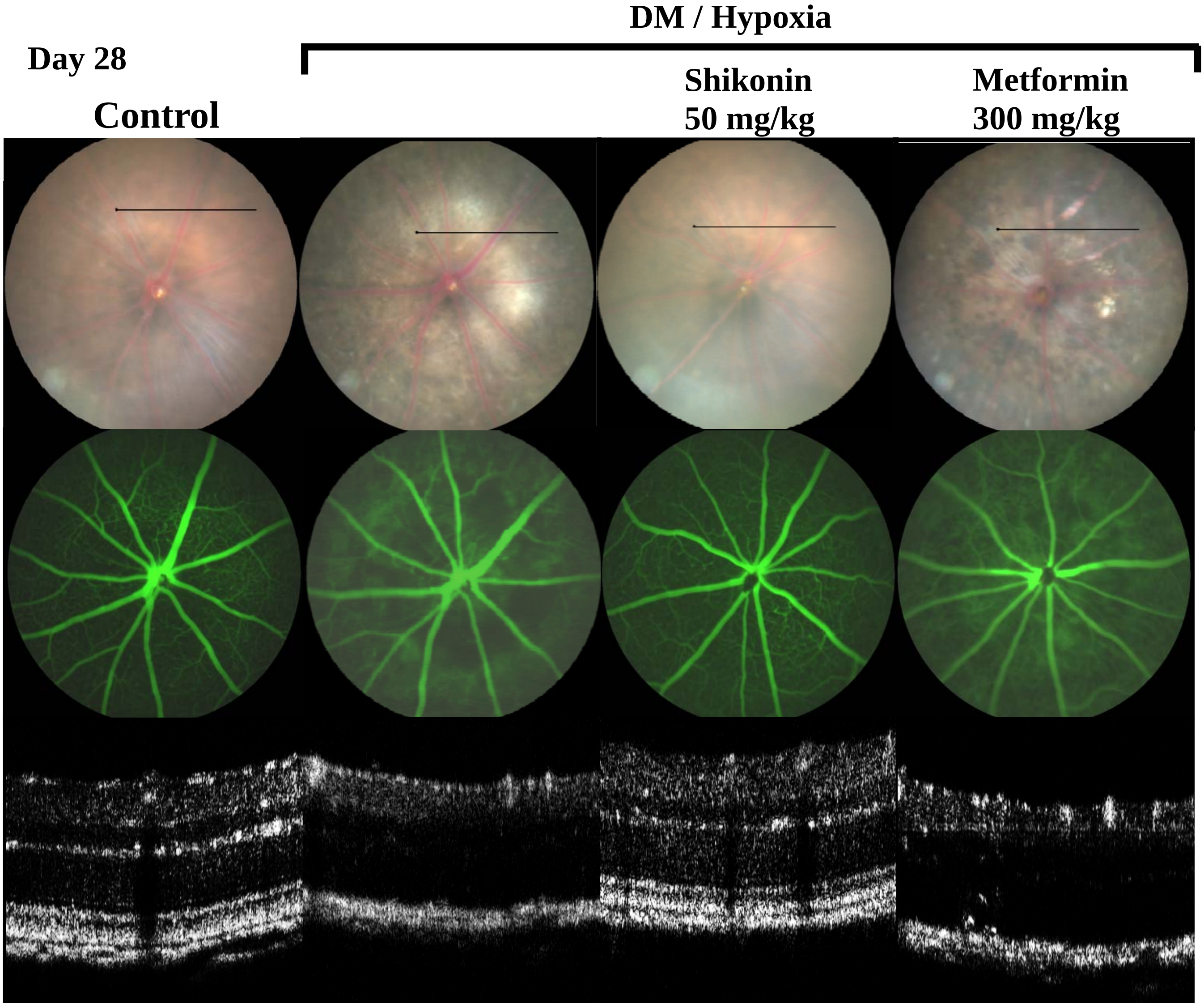

Figure legends

Supplementary Figure S1. Comparison of Metformin and Shikonin in DM/Hypoxia mice

STZ-induced diabetic mice randomly divided into three groups were maintained in a whole-body hypoxia chamber and given orally by gavage metformin (300 mg/kg, qd), shikonin (50 mg/kg) or vehicle for 4 weeks along with non-DM control group. (A) Fasting blood sugar (mg/dL) was measured on day 28th. *** $p < 0.001$ compared with control group; † $p < 0.05$ compared with the untreated DM/hypoxic group. (B) SD-OCT, fundus images following Fluorescein angiography were shown from control group, the diabetic/hypoxic group, 50 mg/kg shikonin treatment group and 300 mg/kg metformin treatment group on day 28th.
